# Addressing Cyberscams and Acquired Brain Injury (“I Desperately Need to Know What to Do”): Qualitative Exploration of Clinicians’ and Service Providers’ Perspectives

**DOI:** 10.2196/51245

**Published:** 2024-01-29

**Authors:** Kimberly Ann Chew, Jennie Ponsford, Kate Rachel Gould

**Affiliations:** 1 Monash-Epworth Rehabilitation Research Centre Richmond, Victoria Australia; 2 Turner Institute for Brain and Mental Health School of Psychological Sciences Monash University Clayton, Victoria Australia

**Keywords:** cyberscam, cyberscams, fraud, cybercrime, cybersafety, brain injury, disability, neurorehabilitation, interventions, treatment, qualitative

## Abstract

**Background:**

People with acquired brain injury (ABI) may be more susceptible to scams owing to postinjury cognitive and psychosocial consequences. Cyberscams result in financial loss and debilitating psychological impacts such as shame and mistrust, interference with neurorehabilitation, and reduced independence. Despite these significant consequences, there are no psychological treatments to support cyberscam survivors. There is limited evidence regarding how the current workforce is addressing post-ABI cyberscams.

**Objective:**

This study aims to understand the perspectives and needs of clinicians and service providers in addressing post-ABI cyberscams.

**Methods:**

Overall, 20 multidisciplinary clinicians and service providers were recruited through purposive sampling across Australia. Semistructured interviews explored post-ABI scam experiences and vulnerabilities, treatments and their efficacy, and recommendations for future cybersafety recovery interventions. Reflexive thematic analysis was used.

**Results:**

In total, 8 themes encompassing a biopsychosocial understanding of scam vulnerabilities and impacts were identified: “genuine lack of awareness: cognitive-executive difficulties”; “not coping with the loss of it all”; “needing trust and connection”; “strong reactions of trusted others”; “nothing structured to do”; “financial stress and independence”; “cyberability”; and “scammer persuasion.” Each theme informed clinical recommendations including the need to provide psychological and cognitive support, enhance financial and cybersafety skills, promote meaningful social engagement, and foster collaboration between families and clinical support teams.

**Conclusions:**

The multifaceted range of scam vulnerabilities and impacts highlighted the need for individualized, comprehensive, and targeted treatments using a biopsychosocial approach to enable cyberscam recovery among people with ABI. These findings will guide the development of a co-designed intervention.

## Introduction

### Background

Acquired brain injuries (ABIs), such as traumatic brain injury and stroke, are leading causes of disability, affecting 1 in 45 Australians [1]. People with ABI often experience significant cognitive-communication difficulties, mental health challenges, increased social isolation, and reduced participation in meaningful activities [2,3]. These factors often interact with each other, highlighting the need to understand and address the impacts of biological, psychological, and social (ie, biopsychosocial) processes [4]. To reduce isolation and form meaningful relationships, people with ABI may engage in web and social media use [5,6]. Although there are many benefits to online engagement, cybercrimes, such as scams, are rapidly increasing internationally. In 2022, more than Aus $3 billion (US $1.97 billion) was lost owing to scams in Australia, with 71% increase in scams reported by people with disability in 2021 [7]. Anyone can be scammed; however, post-ABI sequelae including lack of insight and impulsivity may place people with ABI at increased risk [8,9].

Cyberscams are crimes that leverage mass communication tools to defraud people for financial gain [10]. Common cyberscams include investment, dating and romance, false billing, phishing, remote access, threats to life and arrest, identity theft, job and employment, online shopping, and classified scams [7]. Reports have highlighted the digital vulnerabilities of community groups such as adults with disability and young people [11-13]. People with disability are also more susceptible to cybercrimes related to threats to life, arrest, ransomware, other malware, and mobile premium services [7]. Although no study has directly investigated the relationship between cognitive impairment in ABI and scam susceptibility, studies of older adults with mild cognitive impairment and vascular pathology lend some support to this contention [14-16]. There is currently a paucity of established evidence specifically regarding cyberscams or safety and ABI [17,18]. A recent survey of 101 ABI clinicians and service providers found that 53.5% of participants had at least 1 client with ABI affected by cyberscams, with romance scams encompassing the majority, highlighting the need for clinical and research attention toward this matter within neurorehabilitation [9]. Participants described that their current management approaches include psychologist referrals, building awareness, and increasing community engagement, yet they did not endorse any strategy as particularly effective.

Qualitative interviews exploring the lived experiences of people with ABI who have been scammed and close others (COs) revealed a complex scam journey, with multiple significant difficulties extricating from the scam or its impacts [8]. Factors contributing to scams included scam vulnerability, scammer manipulation, reduced cyberscam awareness, financial and psychosocial functioning, and unhelpful or restrictive cyberscam responses from families and services (eg, limiting internet and financial access). Distressing psychological consequences such as shame, fear, and mistrust were often experienced [8,9], similar to the general population [19-21]. These psychological consequences are often debilitating owing to a “double-hit” of both financial and emotional distress [20]. Post-ABI scams have also interfered with neurorehabilitation treatment and reduced independence [9]. Intervention recommendations from those with lived experience included increasing scam awareness; reducing shame through hearing scam stories; and normalizing scams through the provision of education, acknowledging how common scams are, and reassuring survivors not to blame themselves [8].

There is a critical need for evidence-based strategies to address the significant unmet financial and psychological impacts of cyberscams. Cyberscam prevention campaigns have been implemented for the general population [22,23]. However, despite the significant distress experienced by survivors, no known intervention has been identified to address the psychosocial impacts of cyberscams. Furthermore, people with ABI who are scammed may require tailored interventions, which account for their cognitive, emotional, and functional impairments. Our group recently co-designed CyberAbility [17], an ABI-tailored, prevention-focused, cybersafety training program. “Cyberability” refers to the ability to learn and adapt to current and emerging technologies (eg, safe and responsible online use) [24]. Social-ABI-lity, an ABI-specific web-based social media skills training program, has also been recently developed [18]. Nonetheless, there remains a need for ABI-tailored interventions that specifically support psychological recovery after the cyberscam. Interestingly, participants involved in the CyberAbility [17] co-design process experienced it as “an intervention addressing shame” [25]. This suggests potential for supporting scam recovery through sharing stories, peer bonding, and meaningful contribution.

### Objective

Clinicians and service providers play a central role in supporting and providing treatment to people with ABI who have been cyberscammed. Currently, there is limited research investigating the current treatment landscape among people with ABI [9]. Moreover, the secondary impacts of addressing cyberscams on health professionals is unknown. Therefore, this study aimed to qualitatively understand the experiences and recommendations of clinicians and service providers in managing the impacts of cyberscams among people with ABI.

## Methods

### Design

The study had a qualitative design. This paper is presented based on the COREQ (Consolidated Criteria for Reporting Qualitative Research) 32-item checklist [26].

### Ethical Considerations

Institutional ethics approval (Monash University Human Research Ethics Committee; 17984) and informed consent were obtained.

### Procedure

Authors KRG and KAC conducted the interviews between January 2019 and October 2022. Both interviewers were women with at least an honors degree in psychology and were trained in qualitative interviewing and analysis. Interviews were conducted face to face (2/25, 8%) or via videoconference (23/25, 92%). Interviews were audio recorded and transcribed externally or by KAC. Interviews ranged from 37 to 81 minutes in duration. Interviewers kept reflection journals for each interview. An interview summary was sent to participants to ensure that their views were accurately captured and to seek additional feedback. Comments were integrated into the findings before analysis.

### Participants

Multidisciplinary clinicians and service providers were recruited via an existing research database, professional networks, and social media, with invitations to participate sent via email. Eligibility criteria were the following: clinicians or service providers with experience in providing assessment or intervention to adults with ABI, experience in cybercrime vulnerability or supporting an adult with ABI who had been affected by a cybercrime, and working within Australia. Overall, 20 participants were recruited. There were preexisting interviewer-participant relationships (professional colleagues) among 35% (7/20) of the participants. Participant characteristics are displayed in Table 1. Most participants (18/20, 90%) were female, with an average of 18 (SD 9) years of experience and a bachelor’s degree or higher qualification. Most (13/20, 65%) were clinical neuropsychologists and occupational therapists working in community settings.

**Table 1:** Participant demographics (N=20).

Characteristics			Value
Age at the time of interview (y), mean (SD); range			43.89 (0.54; 25-63)
**Sex, n (%)**			
	Female	18 (90)	
	Male	2 (10)	
**Highest level of education, n (%)**			
	Doctoral degree	6 (30)	
	Master’s degree	9 (45)	
	Graduate diploma	1 (5)	
	Bachelor’s degree	4 (20)	
Experience at the time of interview, mean (SD; range)			18.43 (9.99 y; 8 mo-42 y)
**Discipline, n (%)**			
	Clinical neuropsychology	9 (45)	
	Clinical psychology	2 (10)	
	Occupational therapy	4 (20)	
	Psychologist with no endorsement	2 (10)	
	Service provider	3 (15)	
**Work setting, n (%)**			
	Community or private practice	16 (80)	
	Hospital	1 (5)	
	Forensic department	1 (5)	
	Not-for-profit organization	2 (10)	

### Measures

An initial semistructured interview schedule was designed to explore client cyberscam incidents, prevention strategies, resources, confidence in personal cybersafety, and skills in addressing cyberscam impacts with clients. Following a reflexive design, the interview schedule was revised iteratively based on the generated themes, gaps, and areas of interest identified during the interviews. Additional questions were related to current and future interventions, scam and intervention engagement, and recommendations for intervention content and structure. Refer to Multimedia Appendix 1 for the interview schedules. Of the 20 participants, 5 (25%) participants completed the initial interview schedule only, 5 (25%) completed the initial interview and a second interview covering the additional questions, and 10 (50%) completed a single interview covering all questions (ie, a total of 25 interviews were conducted with the 20 participants). Participants’ demographics were obtained using a brief questionnaire.

### Data Analysis

Descriptive statistics for participant demographics were summarized using Microsoft Excel (2019). Interview transcripts were analyzed using the 6-phase thematic analysis by Braun and Clarke [27] to identify themes reflecting the perceptions about client experiences and intervention recommendations. A nonpositivist [28] and nonlinear format [29,30] was used to analyze themes. First, data familiarization was conducted, where data were repeatedly reviewed. Second, initial codes were generated inductively. Codes were cataloged using NVivo (version 12.5.0; Lumivero) [31]. KRG coded the first 36% (9/25) of the interviews, and KAC coded the remaining 64% (16/25) of the interviews. Acknowledging that reflexive thematic analysis does not require cross-coding [32], 8% (2/25) of the transcripts were cross-coded by KAC as part of her training. Excellent intercoder reliability was found (mean Cohen κ=0.83; range 0.31-1) [31,33]. In total, 3 codes with Cohen κ<0.50 were reviewed by both coders [34], and consensus was achieved through discussion. Third, codes were classified into high-order themes following repeated review with all authors. Provisional themes were discussed and refined 7 times among all authors. Themes were visually mapped using Miro (Realtime Board, Inc), a collaborative whiteboard [35]. Fourth, the thematic map and themes were checked against the original transcripts and refined to ensure accuracy. Fifth, once consensus was achieved, themes were defined and labeled. Sixth, a written conceptualization of the findings was prepared, and representative quotes were selected.

## Results

### Overview

In total, 20 clinicians and service providers described 34 cyberscams experienced by their clients with ABI who had predominantly moderate to severe traumatic brain injury or stroke. Most cyberscams reported were romance or friendship scams (17/34, 50%), with other scam examples including buying and selling, prize, service provider, and investment scams. Scam impacts included near-misses to significant financial loss (eg, Aus $250,000 [US $168,801]; “all his savings”) and psychological distress. Scam survivors were also placed in personal danger and experienced sexual safety impacts. Striking romance scam examples included a woman who traveled to Africa alone, requiring rescue by the Australian Federal Police; a man who was sexually extorted; and a woman living in a 24-hour accommodation who was taken advantage of by registered sexual offenders she met on the web, resulting in childbirth.

**Figure 1:**
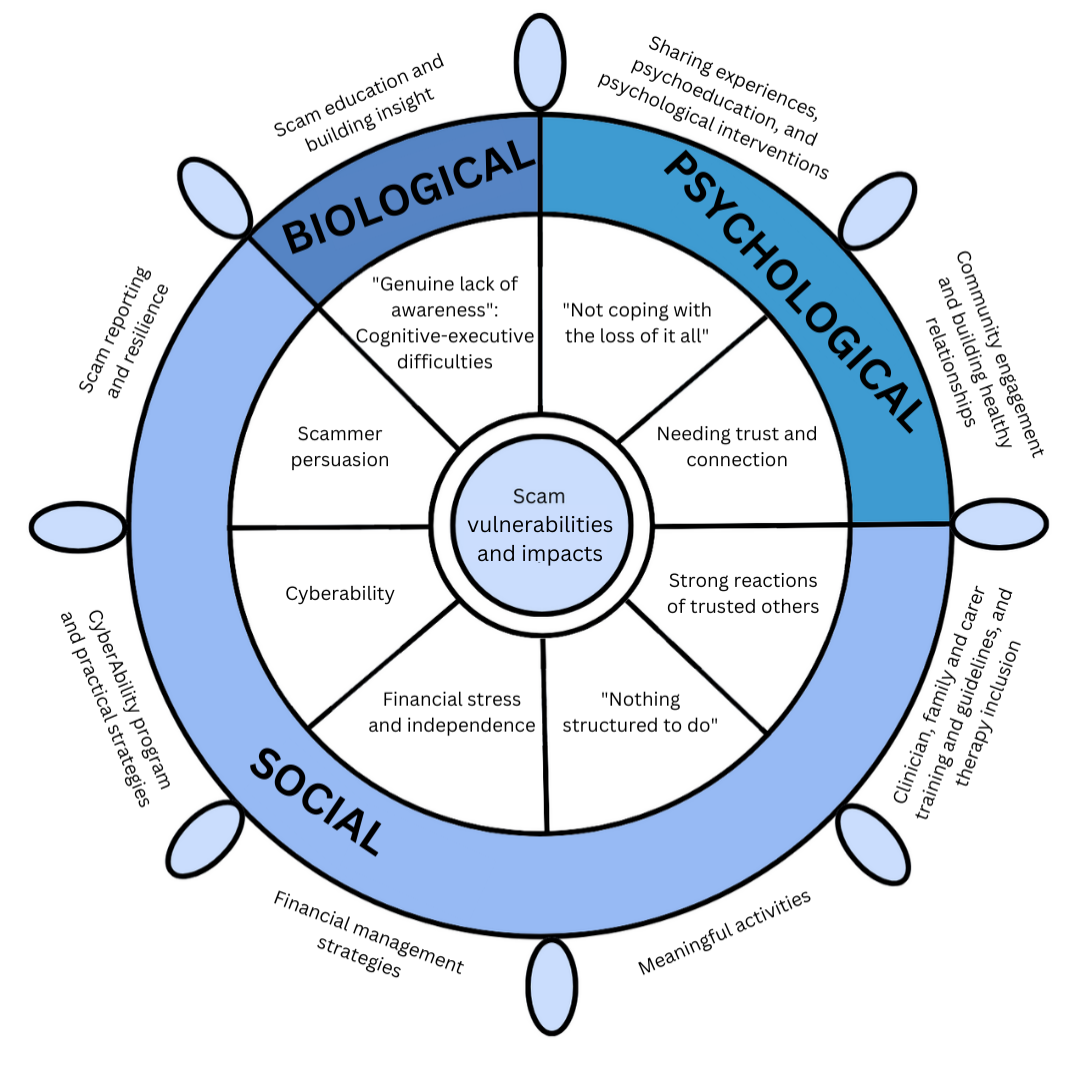
Thematic map of clinicians' and service providers’ perspectives.

Overall, 8 interlinked themes relating to cyberscam vulnerabilities and impacts on people with ABI were generated by the researchers through reflexive thematic analysis (Figure 1). Cyberscam vulnerabilities and impacts were organized into biological, psychological, and social aspects and visually displayed by a ship’s helm to represent the interrelationships of these themes with equal influence on the conceptualization of survivors’ scam experiences. ABI-related cognitive impairment underpinned vulnerability and impacts by reducing the individual’s ability to recognize and protect themselves from scams. Furthermore, ABI was associated with impaired coping capacity and reduced ability to navigate important relationships with support people concerned about the scam. Owing to ABI, support people commonly implemented restrictive practices that exacerbated rather than mitigated cyberscam risks and impacts. Theme-specific intervention recommendations, shown in the outer ring of the helm, are described separately after exploration of the thematic results.

### “Genuine Lack of Awareness”: Cognitive-Executive Difficulties

#### Overview

ABI was a key biological factor underpinning vulnerability to cyberscams. Specifically, participants highlighted that cognitive-executive impairments associated with ABI increased cyberscam vulnerability owing to lack of insight, difficulties in generalizing between experiences, and impaired memory for vital information. Although lack of insight occasionally protected against the emotional impacts of cyberscams, people with ABI remained vulnerable to future cyberscams owing to difficulties in learning from mistakes.

#### Cognitive Vulnerability

Most participants (15/20, 75%) noted that anyone can be scammed; however, “if we’re all vulnerable, then...someone with an ABI is even more vulnerable” (participant 13). Specifically, all participants (20/20, 100%) identified executive dysfunction as a significant cyberscam vulnerability, including lack of insight; cognitive rigidity; and impairments in problem-solving, reasoning, decision-making, understanding complex information, generalizing, and learning from mistakes. Executive deficits and reduced insight placed some people with ABI in dangerous situations owing to difficulties in identifying red flags and appreciating both real and potential risks—an occupational therapist described the physical danger her client faced when she traveled to Africa to meet the scammer she thought was her boyfriend:

She came so close to losing her life in many ways or being assaulted or she was in a hotel room on her own for a week with no food.... She took $50 Australian with her, she caught taxis on her own...but still, she doesn’t seem to have that level of severity of the concern at all of that situation...she’ll be, “Really? I didn’t see that.”... I think that’s just genuine lack of awareness because of her brain injury.

Participant 2

Executive difficulties also underpinned behavioral dysregulation. More than half of the participants (11/20, 55%) shared that their clients with ABI engaged in impulsive and risk-taking behaviors. People with ABI were left vulnerable to scammer tactics (eg, time pressure) owing to “inability to regulate her behaviour in the moment when she sees an instant reward” (participant 18).

Brain injury–related reductions in complex communication skills such as comprehension of implied information, judgment, and self-monitoring increased risks during online discourse:

It’s a lot easier to misinterpret what you say.

Participant 5

In combination, these cognitive-executive, behavioral, and communication difficulties underpinned by ABI resulted in increased vulnerability to being repeatedly scammed and reduced self-awareness of this susceptibility.

#### Barriers to Recovery

Participants described that concrete and inflexible thinking presented barriers to cyberscam recovery as it took time and additional support “because of his brain injury to put two and two together” (participant 15). Moreover, disbelief and denial, in conjunction with the grooming by scammers, made it difficult for clients to accept the scam and engage in intervention as they still believed that they were “getting special scam treatment” (participant 2). Cognitive difficulties of impaired memory and reduced insight interfered with intervention effectiveness:

You weren’t gonna be able to do any kinds of therapy with him, any intervention of that nature. He just wouldn’t have been able to...hold on to it, and...believe that he needed it.

Participant 17

For those who accepted the need for cybersafety practices, memory and executive difficulties reduced an individual’s ability to learn from mistakes and use strategies when responding to high-risk cybersafety situations in real time, contributing to cyberscam vulnerability and re-engagement:

He remembers that it’s something he should be doing so he does hang onto that information...when he’s prompted...But in the heat of the moment, that doesn’t necessarily come into his mind as the first thing he’s gonna do.

Participant 8

#### Short-Term Protection

Interestingly, 20% (4/20) of the participants shared that lack of insight initially appeared to protect people with ABI from negative emotional impacts of the cyberscam. However, as a result of the absence of a negative reaction to the scam, they were less likely to learn from their experiences, contributing to repeated exposure to scams:

She recovered from [a little bit of shame] pretty quickly. But she also re-engaged with another scammer. So she wasn’t down for long. It’s almost like – yes, her heart was broken, but then she kind of went back and found someone else.

Participant 9

Nonetheless, preexisting psychological difficulties, in combination with limited social connection and cognitive impairment, increased scam susceptibility:

His isolation and depression...combined with his TBI [traumatic brain injury] just left him really vulnerable.

Participant 17

This was compounded by the trusting and generous nature of participants, who empathized with the convincing stories told by cyberscammers as part of their grooming techniques, which made it hard for survivors to accept the scam:

He still reflects on how sorry he felt for her [cyberscammer], how difficult her life was, how he felt he could offer her a stable and good life.

Participant 16

### “Not Coping With the Loss of it All”

#### Overview

Psychological difficulties before the scam, combined with reduced cognition, were reported to increase cyberscam vulnerability. All participants (20/20, 100%) also identified significant psychological repercussions of the scam among people with ABI, particularly, shame, depression, anxiety, and denial. Clients who found it hard to accept the cyberscam often re-engaged with the same scammer:

That scam had gone on for many years...and he, even at the time that I exited working with him, was still not convinced that story wasn’t true.

Participant 16

Debilitating psychological impacts also interfered with treatment and rehabilitation.

#### “Life Plummeting to New Depths”

Several significant psychological impacts were identified by participants where their clients were “psychologically, emotionally, financially abused, and it can lead to suicide” (participant 9). Depression, anger, embarrassment, mistrust, shame, and guilt were the most common psychological impacts reported:

She was angry. She’s like, “I’ve been so stupid. I should never have done this. I can’t believe I’ve been scammed.”

Participant 3

For those embroiled in romance scams, losing the relationship with the scammer often resulted in depression:

To think you’re going to get a relationship and you’re not is hugely devastating for our client group.

Participant 4

Participants expressed that their clients felt a loss of hope and confidence “in her ability to make friendships and more intimate relationships” (participant 10). For some clients, getting scammed facilitated awareness of differences in cognitive and functional abilities after the injury. However, increased insight into postinjury function also exacerbated emotional distress:

He spent a lot of time crying.

Participant 15

The injustice of the scam was particularly difficult to cope with:

Frustration...he seemed to struggle with managing his emotional response to wanting to help, feeling like a good guy...like he’s doing the right thing for someone and then yet having all this other bad stuff happen as a result, and his life plummeting to new depths.

Participant 16

Anger was directed toward family and friends:

He’s got anger with his mum, which he never would have done before, and with a friend that maybe hasn’t called him back in time.

Participant 15

People with ABI also felt “silly...like an easy prey...robbed him of his pride” (participant 11). Anger owing to being scammed also contributed to broad escalations in challenging behavior:

Like he might grab them on the jugular and do stuff to them. Strangle them.

Participant 11

Some people with ABI attempted to retaliate against the scammers by re-engaging with them. Despite this appearing to be a reasonable motive, people with ABI increased their risk of another cyberscam in the future as “[scammers] are still keeping you on the line” (participant 16).

#### Extreme Mistrust

For some people with ABI, anger following the recognition of the cyberscam led to scam disengagement and heightened awareness of cyberscam red flags. However, cognitive difficulties related to ABI resulted in overgeneralizing from the online scam experience into everyday life, becoming extremely mistrusting and “cutting off other people that were legitimately his friend...because he’s worried about people ripping him off now” (participant 15). Difficult experiences in reaching out to formal and informal supports contributed to additional distress and relationship impact for scam survivors with ABI. Reporting the scam to services such as the police and the resultant unhelpful outcomes exacerbated their “lack of confidence in engaging with services” (participant 10). In contrast, clients who did not accept that they had been scammed were angry with the support people’s attempts to increase their awareness, contributing to a significant “level of mistrust with all services that were trying to help” (participant 1). A psychologist reported that by revealing their client’s scam, their client was “momentarily angry that I ruined her friendship” (participant 12). Relationship breakdowns with family members and friends were common as “he’s got anger with his mum, which he never would have done before” (participant 15). These experiences highlighted the compounding effect of cognitive difficulties (ie, lack of insight and overgeneralization) and psychological factors (eg, anger and loneliness) on important social relationships in the person’s life.

#### Intervention Roadblocks

Psychological responses to the scam acted as major barriers to intervention by interfering with problem-solving to seek support:

The guilt and shame is so much that he’s not even thought, “Hang on, I should go to the police. I’ve been ripped off.”

Participant 13

Shame, in particular, served as an obstacle to help seeking from family owing to fear of judgment:

That kind of embarrassment, and the fact that he didn’t want the son involved in this process much when he probably would have been a good support for him.

Participant 13

Significant anger and challenging behaviors triggered by discussing the scam prevented clinicians from directly addressing the scam during their interventions:

He would ramp up his agitation so then you’d have to switch topics, otherwise, he would get quite nasty.

Participant 15

When able to be provided, support from trusted others through reassurance and counseling curbed some negative emotional impacts. In contrast, restrictive practices and increased supervision were considered to have undesired effects on independence and relationships through deteriorations in trust:

[His mother] still had a sense of protectionism...she was checking his bank statements...the biggest impact was the fact that she didn’t trust him anymore.

Participant 16

### Needing Trust and Connection

#### Overview

Social factors increased the vulnerability to cyberscams and interacted with the life-changing impact of ABI. Psychological vulnerability to scams was closely tied to a strong desire for trusting romantic relationships and social connection as people with ABI “wanted the white picket fence family future that he’d always dreamt of” (participant 16). However, presence of a trusting and supportive relationship did not protect against cyberscams for people with ABI if their caregivers were not cyber aware.

#### Strong Desire for a Trusted Relationship and Intimacy

Although participants reported that some of their clients with ABI were aware of romance scam red flags, their strong drive for a relationship outweighed the potential for financial or emotional loss:

[The client] agreed not to do it again. But he...desperately wanted a relationship and he saw [the scammer] as the only solution.

Participant 7

This was often driven by social isolation and a “deep loneliness a lot of clients have that they just wanna be loved [and] have kids” (participant 8). People with ABI were often isolated, had difficulty in integrating into their community, and lacked proximal social support, increasing their motivation to search on the internet for connection:

She had to separate from her husband after a brain injury...had a string of relationships that didn’t work out...a lack of meaningful activities in the community and few social supports so a lot of her world was online.

Participant 2

These same drivers for connection were also reinforcing for family members and carers, who encouraged people with ABI to engage in online relationships. Compounded by their own limited cybersafety knowledge, family members were described as facilitating scam engagement:

Mum probably saw these as potential legitimate relationships...she would get angry with him and say, “Why are you being so rude to these people who are trying to be friends with you?”

Participant 15

#### Clinician-Client Relationship

The presence or absence of a strong therapeutic relationship was an instrumental social factor in addressing cyberscam vulnerability and interventions for people with ABI. People with ABI who were isolated and had limited clinical and support services were more susceptible to the grooming and psychological influence of scammers. They also lacked support from trusted therapists in identifying and managing cyberscam impacts. Not having a strong, trusted clinician-client relationship was a barrier to treatment effectiveness as “if they don’t trust you, they just don’t tell you anything” (participant 9). In contrast, interventions were considered to be effective when clinicians had a good awareness of their clients’ needs and an established therapeutic alliance:

I knew her and how far I could push her.

Participant 12

Nonetheless, it was acknowledged that building a trusting working relationship was more challenging for clinicians who engaged with clients in a once-off setting (eg, conducting neuropsychological assessments).

### Strong Reactions of Trusted Others

#### Overview

The combination of reduced insight and difficulty in identifying red flags, coupled with a strong desire for social connection kept people with ABI hooked onto the scam. This often led to significant financial loss, prompting strong reactions from clinicians and caregivers. Although some were supportive and involved, others were unsupportive and angry. Participants felt frustrated and helpless and lacked confidence in their own capacity to support their clients’ recoveries from cyberscams.

#### “Tumultuous Relationships”

For people with ABI who commonly experienced postinjury social changes, “tumultuous relationship[s]” (participant 4) with family and other caregivers interfered with cyber awareness and seeking support. These relationships made it difficult for clients to share what was happening in their lives:

He’d only just resumed contact after they’ve had a falling out...he wouldn’t consent to me contacting his son for an informant history.

Participant 13

Arguments between people with ABI and caregivers were common after the cyberscam—“huge fights with his family” (participant 4) and “breakdown of friendship” (participant 10). This was attributed by participants to the lack of insight and inability to accept the cyberscam among the people with ABI, combined with caregiver’s blame of the person with ABI for the financial loss, reinforcing feelings of shame. People with ABI therefore lost important social supports that could serve as cyberscam-protective factors. Existing challenging behaviors also deteriorated relationships as difficulties with self-regulation “means that the discussions escalate to a point where you can’t reasonably talk about it [and] have helpful discussions” (participant 3). Some caregivers therefore limited their interactions to avoid conflict. In contrast, some clients had supportive and reassuring caregivers helping them through the process by supporting online participation access and trying “to maintain that normal home life rather than a knee-jerk reaction” (participant 10).

#### Clinician’s Distress

Participants described the experience of their client being scammed as “heartbreaking” (participant 2), noting that it “lingered for a while but more so than any other devastating thing that happens to our clients” (participant 6). The severity and unexpected nature of the scam situation and clients’ significant financial, resource, and independence losses contributed to these persistent feelings. Participants working on established rehabilitation goals, expressed anger as “it takes you away from the pathway and the goals you would have been working towards” (participant 3). Cyberscam re-engagement contributed to feelings of frustration as “we’d built this man’s independence really significantly...I didn’t wanna see that disappear” (participant 7). Nevertheless, some participants reported addressing cyberscam experiences as an opportunity for professional development and change in clinical practice:

I ask about online relationships now, which I never used to.

Participant 9

### “Nothing Structured to Do”

#### Overview

Participants noted that unstructured and purposeless time meant that people with ABI were spending more time on the internet and were more socially isolated, highlighting increased cyberscam vulnerability. Loss of funds and restrictions in self-management of finances owing to the scam often resulted in reduced capacity to participate in meaningful activities and engage with the wide community.

#### Reduced Meaningful Engagement

Unstructured time exacerbated the vulnerability to scams through extensive online engagement, such as a neuropsychologist’s client with ABI who had an “addictive personality and...was using his iPad for a lot of his waking hours” (participant 3). Although it served a purpose as a form of community engagement (“it was filling a gap in her social isolation” [participant 2]), more screen time increased clients’ cyberscam exposure. Even people with ABI who had structured daily activities were vulnerable and likely to engage in cyberscams during evenings and nights:

It’s very hard to fill those gaps, from a therapeutic, rehab, work perspective.

Participant 7

Being isolated during this time frame increased cyberscam vulnerability owing to ABI-related cognitive impairments (eg, reduced insight into scams and impaired memory for red flags), which was compounded by other factors such as unsupervised access to technology and desire for relationship.

Following the scam, participants described that people with ABI became less likely to engage in meaningful activities for several reasons. For some, this related to loss of self-confidence in navigating social events and meeting new people:

Because of the risks of doing the wrong thing – especially if it’s a relationship scam – trusting people again.

Participant 19

For others, infatuation with the scammer reduced interest in meaningful participation such as “Men’s Shed or...a woodwork group...because [the scam] was his focus” (participant 3).

Scams that resulted in significant financial loss also led to the loss of ability to afford leisure activities and led to clients having excessive unfilled time.

### Financial Stress and Independence

#### Overview

Participants identified that clients experienced financial stress and changes to independence before and after the cyberscam. This was often paired with the strong reactions of trusted others who sometimes turned to restrictive practices as a way of financially protecting their loved ones. Being under financial administration before the cyberscam often left clients without sufficient financial self-management skills. Some clients had disposable income and financial resources to accommodate scammer requests; wealthy clients ended up with significant financial losses. Some clients who independently handled their financial affairs were deemed to have lost their financial decision-making capacities in the face of the scam.

#### Limited Financial Knowledge and Independence

Cognitive impairments after ABI often reduced financial literacy. More than half of participants (11/20, 55%) reported that their clients received financial oversight (eg, administration orders) or informal support. Financial administration alone, however, was insufficient to prevent cyberscam engagement:

Even though his funds are protected...he had enough access to that amount of money [$500] to transfer that.

Participant 5

Nonetheless, restricted financial access limited significant financial loss to scammers:

He couldn’t give her the money because of this “stupid administration order” and [financial trustee].

Participant 3

Although financial loss was significantly reduced, financial administration orders were often described as paternalistic and left clients without appropriate financial management skills as “he wouldn’t have developed those skills in managing his own money and setting up those practical things” (participant 13).

Some people with ABI arranged to have financial administration orders removed at the behest of the cyberscammer. Attempts by people with ABI to demonstrate their ability to manage their finances independently interfered with client-clinician relationships as clients hid key information:

He felt that he needed to prove that he wasn’t spending his money in a poor way, that his decision-making wasn’t bad, so he wasn’t always keen to let us know what he was spending his money on.

Participant 4

Moreover, restricted financial access increased clients’ desire for control in other aspects of their life. Romance scam vulnerability was increased as clients were “more driven towards this goal [finding someone] because it’s the only thing he thinks he could independently do...that’s not restricted” (participant 3).

#### Loss of Finances and Decision-Making Capacity

Cyberscam engagement resulted in the loss of finances and important resources. Several participants (17/20, 85%) reported that their clients had lost all of their savings, inheritance, and compensation payouts:

Sold all his shares...AUD$250,000 [USD $168,801] that went overseas.

Participant 6

Wiped out most of his savings.

Participant 14

A really large financial compensation payout...all gone.

Participant 16

Financial loss contributed to significant loss of independence and participation in meaningful activities, which often exacerbated psychological distress. A client “couldn’t afford to do activities that he previously...enjoyed because his resources were completely depleted” (participant 16). Examples include the inability to pay rent, pay for car registration, and continue living their current lifestyle (eg, traveling overseas and going to concerts and football games). The reduced ability to engage in these important social and leisurely pursuits significantly affected the quality of life and increased social isolation.

Financial independence was at risk, with some clients requiring a review of their decision-making capacity after the scam:

He was so clearly hugely vulnerable and unaware of how vulnerable he was that I actually made the recommendation that he probably shouldn’t even be managing his disability pension.

Participant 17

Loss of financial capacity often left clients feeling vulnerable about “losing his independence separate to the scam experience” (participant 16). For clients with limited insight into postinjury impairments, losing financial access and dignity to take risks resulted in emotional distress:

The depression...didn’t come from being scammed. It came from quite the opposite, almost like he...feels like it’s his right to have access and leave himself open to those things.

Participant 17

In contrast, some participants, albeit a minority, reported little to no financial loss for their clients. Restricted access to funds, carer scam knowledge, and supervision contributed to “near-misses”:

His carer very quickly identified that he was having [scammer] requests.... He just didn’t have the access to money to be able to send that through.

Participant 10

### Cyberability

#### Overview

Participants described that online engagement was seen as a means to fill the gaps of reduced meaningful participation and subsequent social isolation for people with ABI; however, basic levels of cybersafety knowledge and awareness was required. Participants provided examples of their own cybersafety experiences such as awareness of social media privacy settings, blocking and deleting scam calls or emails, and being skeptical of unexpected requests for information. Participants reported that reduced levels of cyberability in their clients and themselves increased cyberscam susceptibility. In contrast, adequate cyberability might also create a false sense of security and overconfidence in online behavior.

#### Limited Technological Exposure and Knowledge

Limited cyberability presented as a lack of understanding of the internet and computers. Preinjury factors including socioeconomic disadvantage or incarceration led to limited knowledge and exposure to technology:

Coming out of correction orders, they haven’t had any technology for five to ten years.

Participant 1

Lack of technological exposure also reduced awareness of personal privacy protection, which was perpetuated by ABI-related cognitive impairments:

She wasn’t a high internet user prior to her brain injury, now all of sudden she found herself on social media sites...using an iPad...and really has no awareness of protecting her privacy, much more than the general person would experience.

Participant 2

Lack of direct observation by clinicians made it challenging to ascertain whether people with ABI participated in safe online behavior, even those residing in 24-hour supported accommodation. With limited cybersafety awareness, unsupervised access to technology was concerning to participants as “you wouldn’t have a clue what they’re up to” (participant 5). For people with ABI who were dependent on others, having trusted relationships protected them from cyberscam attempts as “he has somebody in the room with him...they just happened to notice what he was doing and intervened” (participant 10). However, this was with the caveat that caregivers had strong cyberability:

I’ve had stories of people whose support worker has clicked the [scam] link for them.

Participant 16

In contrast, sound technological knowledge and online independence was considered to be insufficient in protecting against cyberscams as it created a false sense of security:

I think perversely his technical prowess with IT stuff...and obviously scammers rather exploit that vulnerability as well.

Participant 8

This was compounded by the cognitive-executive difficulties experienced by people with ABI who “...don’t have the ability to filter and work out what’s safe and what’s not safe” (participant 11). Individuals might therefore appear overconfident about their cybersafety skills, potentially masking their vulnerability, and this results in low motivation to engage in scam prevention education.

Overall, 25% (5/20) of the participants spontaneously reported that they had used the web-based CyberAbility [17] training program and found this to be helpful in improving the cybersafety skills of their clients with ABI, with a particular benefit of reducing scam stigma and improving scam prevention:

We did the CyberAbility, it’s like, “Yes, it’s happened to lots of people!” and it’s never happened again.

Participant 15

The program helped to facilitate discussions with clients as it “allowed me to have more conversation with her about this than I ever have...because it’s a more formalised way” (participant 18).

#### “Anti-Facebook”

Some participants noted a change after the cyberscam where clients stopped using social media, emails, and internet owing to generalized mistrust of technology. Despite this conferring protection against future cyberscams, this negatively affected their social communication, as some participants expressed that “it’s very excessive because she’s reluctant to go on social media...emails” (participant 10). A participant shared that although their client restricted his digital engagement, this was only short term as “he likes...the connection to the world through his computer and the convenience. But he will not go back on to any type of online banking” (participant 20). This may negatively affect function with reduced accessibility to services, transaction delays, and potential security concerns. Online re-engagement also did not return to prescam patterns for many clients.

### Scammer Persuasion

#### Overview

Scammers leveraged the limited levels of cyberability among people with ABI and their desire for a relationship to keep them hooked onto the scam. Participants described that scammers used tactics such as love bombing, personalization, constant reinforcement, and isolation to keep the clients hooked onto the cyberscam:

The person online was very nice, made her feel very happy and attractive and confident.

Participant 10

Coupled with cognitive-executive difficulties, people with ABI therefore had difficulty in disengaging from ongoing contact with the scammer and resisting subsequent scam attempts.

#### “Sucked in ‘Cause it Sounds So Convincing”

Participants identified that scammers used love bombing strategies to groom and manipulate clients by making them feel loved and wanted and promising a future together. Scammers used clients’ trusting personalities to keep them hooked:

It was a person saying, “You’re amazing, you’re capable, you’re worthy of being loved,” and he [client] was saying, “that is attractive.”

Participant 16

Scammers also manipulated clients’ generosity and fabricated common experiences, such as trauma:

There is this Aussie spirit of helping a mate when your chips are down, coupled with...that sense of, “We’ve got this shared trauma, let’s get together and we’ll be stronger for it.”

Participant 16

Scammer techniques were increasingly sophisticated and personalized (“he’d say he had similar experiences and it was all just too perfect” [participant 9]), therefore reinforcing the desire for connection. Scammer strategies evolved with clients’ patterns of online engagement, which made it difficult even for clinicians to identify the red flags and recognize the scam:

As a therapist, I get sort of bit sucked in too ‘cause it sounds so convincing.

Participant 8

#### Relentless Pressure

Participants described that constant reinforcement and availability was a grooming tactic that scammers used to create positive experiences for people with ABI. The relentless requests for money made it difficult for clients to disengage:

He would express a lot of love for her...then it started to be scattered through quite often, “What about the money, babe? What about the money?”

Participant 3

The grooming was so effective that some clients continued engaging with the scammer and “would confide less in them [family]...because most of his life was thinking about her [scammer]” (participant 3). Scammers isolated people with ABI from their social and support networks and reinforced the scam relationship, with participants noting reduced client contact “because they [scammers] would speak negatively of anyone she trusted and was close to...she became more and more separate from her relationships” (participant 9). People with ABI ultimately trusted the scammers instead “because he thought it was everybody else that was doing the wrong thing, not her [scammer]” (participant 16). Most people with ABI were only able to disengage from the scam when scammers stopped contact or when participants provided education or implemented strategies.

### Treatment Recommendations

#### Overview

The significant cyberscam vulnerabilities and impacts experienced by individuals with ABI, identified through each of the 8 themes, warrant the need for targeted interventions. Our participants provided specific cyberscam recovery recommendations to address each of these factors. Given the multifaceted aspects of cyberscam vulnerability and disconnection between knowing and doing, which is common for people with ABI, a multicomponent biopsychosocial approach to interventions was considered to be necessary.

Participants recommended guidelines such as “a clinician toolkit” (participant 8), with clear “referral pathway or resources” (participant 13), especially for clinicians working in assessment roles. Professional development capacity building workshops were suggested:

More extensive training around this concept for the rehab community generally.

Participant 7

Clinicians who attended previous CyberAbility workshops had implemented these learnings into their practice, improving cyberscam awareness among people with ABI, which reduced cyberscam engagement and financial loss:

...That awareness is there...those are part of our conversations we have with [clients] in preventing it.

Participant 11

Participants highlighted the importance of working in multidisciplinary teams as it “sped up the process a bit” (participant 15).

#### Need to Build Awareness

Participants described that building awareness was key to understanding and increasing scam knowledge. Given the cognitive-executive vulnerabilities to cyberscams among people with ABI, scam education needs to be provided in a tailored way as “people are not going to accept interventions until there’s some self-awareness or ownership” (participant 20). Examples included scam education (eg, red flags) and positive behavior support strategies to challenge the veracity of beliefs about the scammer (eg, “Your Theory is that this is your boyfriend, My Theory is that this is a scammer”).

#### Normalization and Psychological Interventions

Most participants (15/20, 75%) recommended psychoeducation and cyberscam normalization to reduce the psychological impacts experienced after the cyberscam. People with ABI could share and listen to lived experiences of others who had been scammed as “maybe this is a safe place and I can say what’s been on my mind that I maybe was wanting to tell someone but hadn’t felt brave enough” (participant 16). Mood support drawn from cognitive behavioral therapy and acceptance commitment therapy approaches were also recommended.

#### Trusted Person in the Community

Reduced everyday social interactions for people with ABI also required targeted input to establish alternative pathways to check if something is a scam in real time. Participants recommended having therapists as a consistent ally and engaging a trusted person in the community (eg, lived experience experts and guardians) for this role. This could reduce clients’ cyberscam engagement and potentially reduce social isolation—for example, regular therapy sessions that emphasized building rapport and trust and teaching people with ABI how to establish healthy relationships. Strong therapeutic rapport and trusting relationships within the broad support team also facilitated positive discussions about scam situations:

Even though he didn’t like or agree necessarily with some of the conversations and their angle, he liked them as humans.

Participant 16

Having trusted relationships in the community helped to reduce social isolation by increasing community engagement such as “organising Bingo, social events, raffles, and the responsibility of looking after the money involved” (participant 20). Workshops for caregivers were also recommended to increase the awareness and understanding of cyberscams and ABI. Involving caregivers whom clients “can do homework with” (participant 17) could also ensure intervention sustainability. Nonetheless, some participants were cautious about including caregivers to protect their client’s privacy.

#### Cyberability Practical Strategies

Other recommendations to address social factors included practical strategies and skill building. Most participants (18/20, 90%) recommended using practical strategies to reduce cyberscam vulnerability and stop scammer contact—for example, reporting the cyberscam to authorities, supporting clients to screen and block numbers or emails, and teaching password and account management. Participants suggested increasing clients’ cybersafety and technological skills and awareness when communicating and socializing with others by “not disclosing all that information until you’ve seen them in person” (participant 11). Some participants also suggested increasing cybersafety through the web-based CyberAbility [17] training program and working on the root cause of the client’s cyberscam engagement (eg, strong desire for a relationship).

#### “Least Restrictive Practice”

Participants noted that financial skill building (eg, budget management) could reduce clients’ cyberscam vulnerability through increasing ownership and understanding of their funds. Although some participants recommended imposing financial restrictions and administration orders, these were mainly considered as a last resort. Most participants (14/20, 70%) suggested operating “from a least restrictive practice” (participant 7) and “getting other measures in place to protect him other than just appointing [a financial trustee]” (participant 13) based on the clients’ stage in the cyberscam journey “because it’s also about the person’s learning...and skill development” (participant 19). Although more restrictive practices such as limiting phone and internet access were also recommended, these were implemented for clients with low awareness or ability to learn from mistakes:

She’s just unable to flexibly change or adapt...you can only concretely manage...restrict her environment and her access to money or phones. That’s the only way she would be 100% safe, which is very challenging.

Participant 2

A strong and trusting clinician-client relationship was important to address clients’ fear of losing independence if they opened up to clinicians about being scammed. Participants also recommended broad monitoring of clients’ rehabilitation progress to facilitate gradual return to self-management of their finances, online activities, lifestyle, and relationships.

#### Meaningful Activities

Community engagement was highlighted as essential to reducing cyberscam engagement and social isolation. A way to do that was to engage clients in meaningful activities to help them integrate and re-engage with others in the community. For instance, volunteering, returning to work, developing hobbies, participation in projects they valued, and sharing lived experiences helped clients find meaning through contributing:

He loves being in the place of giving...He liked being the person to tell his story and help somebody else.

Participant 11

#### Clinician’s Capacity

Clinician-related factors reflected a barrier to treatment when they did not feel confident in addressing the cyberscam with their clients:

It’s very, very complex...it brings in every skill that you have as a clinician to work in this area with the client.

Participant 2

Limited knowledge, experience, and resources reduced confidence:

I felt awful. I had no concept, no idea. I felt silly and dumb...completely unprepared...unaware and unable to support as well.

Participant 14

Moreover, participants were unsure if addressing the scam was within their scope of practice and role:

What is my responsibility?

Participant 6

Some participants shared that their interventions had insufficient intensity and reach. Reduced capacity limited the level of support and expertise that participants could provide to their clients.

## Discussion

### Principal Findings

People with ABI may be more susceptible to cybercrimes owing to their postinjury consequences, specifically, cognitive and psychosocial difficulties [8,9]. This study aimed to qualitatively explore clinicians and service providers’ experiences in managing cyberscams among people with ABI. Reflexive thematic analysis identified 8 themes that described cyberscam vulnerabilities and impacts among people with ABI, reflecting a broad array of biopsychosocial aspects.

Cyberscams are rife within the wide community and population with disability. These scams often target individuals, businesses, or organizations and can lead to financial losses, emotional distress, and other adverse outcomes [21]. Although numerous forms of cyberscams such as investment scams and identity theft exist, most scams reported to affect people with ABI were romance scams. As such, most of our discussion will focus on the vulnerabilities and impacts of romance scams. Romance scams are a low-volume scam but have significant financial and emotional impacts.

Clinicians identified a range of financial, emotional, cognitive, and psychosocial factors that increased the vulnerability of people with ABIs to cyberscams. The vulnerability to cyberscams bestowed by cognitive-executive impairment, social isolation, unstructured time, and trusting personalities was consistent with reports from people with ABI and their COs with cyberscam experience [8] and are commonly experienced after the injury [2,36]. Impaired insight and difficulty in generalizing from past experiences were described by clinicians as key contributing factors to being scammed. Although participants recommended building insight through scam education, it is worthwhile to note that a discrepancy between “knowing and doing” may remain. Specifically, even when people with ABI possess the awareness of how to determine authenticity, limited insight and awareness make executing this challenging in practice, particularly when manipulated by scammer grooming, threats, and time-pressure tactics [37].

Survivors in the general community often report a “double-hit” of both financial and emotional impacts following romance scams [20]. In this study, participants reported significant financial losses experienced by their clients owing to various scams. In ABI, this financial impact is of additional concern owing to great difficulty in reaccumulating funds after the injury, commonly owing to a once-off compensation payout and high rates of unemployment [2]. This was compounded by the psychosocial impact on participation and loss of independence in the daily activities and social pursuits in which their clients engaged previously.

Similar to previous studies in the general community and among people with ABI [8,20,38,39], significant emotional distress and feelings of shame, anger, and loss of trust were experienced after the scam. Taken together, people with ABI may actually experience a “triple-hit” regarding the loss of finances, reduced independence, and emotional consequences after the scam. Whether this is unique to people with ABI or extends to others with disabilities and psychosocial vulnerabilities or the wide population requires verification.

The emerging evidence for a preponderance of romance scams among people with ABI in this and other related studies [8,9] warrants attention. Loneliness and a strong desire for a trusted relationship were described as factors that increase romance scam vulnerability among people with ABI. In contrast, literature evaluating these vulnerabilities in the general population is mixed [40-42]. Scammers typically leverage these vulnerabilities to groom and isolate people from their support networks [43]. Unfortunately, after the scam, people with ABI may experience even great social disconnection owing to conflict with family and reduced financial means for community participation. Therefore, people with ABI may be more at risk of continuing to engage in the current or additional romance scams that offer what is perceived as a meaningful connection. Potentially, interventions that target the development and maintenance of healthy relationships may help to reduce this underlying vulnerability.

Overall, it is essential to recognize the compounding interaction between the psychosocial consequences of ABI and experiencing cyberscams. Cognitive-executive impairments unique to ABI further complicate cyberscam vulnerability, creating a perfect storm that amplifies both vulnerability and the complexity of managing the distress and aftermath of the cyberscam. Crucially, restrictive interventions that initially appeared to be protective (eg, restricted financial control and checking bank statements) had unintended consequences owing to the interaction between the cyberscam and the cognitive aspects of ABI (eg, increased scam awareness resulted in globalized mistrust, affecting relationships). Consequently, a biopsychosocial, flexible, individualized, multidisciplinary, and multicomponent approach to supporting clients to recover from cyberscams was frequently adopted, similar to most other ABI-specific interventions [44-47]. Key recommended strategies included scam education and behavioral modifications (eg, practicing safe password account management and community engagement), consistent with those reported by people with ABI and their COs [8]. Most participants (14/20, 70%) emphasized the use of least restrictive practices to ensure clients’ autonomy and trust. On the basis of our findings, clinical experience, and wide literature, we recommend a biopsychosocial framework that targets the psychological, social, and functional vulnerabilities and impacts of cyberscams, while accounting for the ABI-related cognitive impairments that complicate intervention and recovery. Importantly, digital upskilling of individuals with ABI needs to be prioritized, given the rapid technological advancements and scams that accompany it. Nonetheless, it is also essential for the technological sector to actively address the needs of people with ABI and other disabilities to ensure a safe and more inclusive space for online participation.

Within the context of these treatment experiences and the difficulties in obtaining good recovery outcomes despite implementing routine interventions, significant clinician distress was identified. To the best of our knowledge, our paper highlights original findings of the secondary impact on professionals who support scam survivors. Most clinicians (19/20, 95%) described lacking knowledge, clinical guidance, and capacity to provide support specifically tailored to cyberscams for people with ABI. In addition to feeling underequipped, they also described feeling distressed and frustrated by learning about and trying to support individuals with ABI who had been scammed. Together with the lack of resources, these findings underscore the need to provide clinicians with the desired targeted training and resources to guide practice and increase confidence and capacity to support people with ABI to avoid and recover from cyberscams. Directed toward prevention, the web-based CyberAbility [17] training program was frequently described as helpful in facilitating conversations and educating clients about cyberscams and had resulted in several examples of scam avoidance. The utility of this approach in prevention and treatment requires additional evidence.

Although we captured a broad range of perspectives regarding the treatment landscape across disciplines in Australia, given the global threat of cybercrime, future studies could explore international experiences in supporting people with ABI to recover from postcyberscam impacts. The generalizability of these findings beyond ABI to other disability and vulnerable cohorts also requires examination.

### Conclusions

To the best of our knowledge, this is the first study to qualitatively explore the perspectives of clinicians and service providers in addressing cyberscam experiences among people with ABI. We identified a framework to assist in understanding the vulnerabilities and impacts of cyberscams, which aligns with a biopsychosocial model. The framework guides the design of cyberscam interventions for people with ABI, which are individualized, comprehensive, and targeted to biopsychosocial factors. These findings are now being used to co-design a cyberscam psychological recovery intervention with people with ABI.

## Appendix

Multimedia Appendix 1 Interview schedule.

## Abbreviations

ABI: acquired brain injury
CO: close other
COREQ: Consolidated Criteria for Reporting Qualitative Research
