# Supplementary material for: Addressing Cyberscams and Acquired Brain Injury (“I Desperately Need to Know What to Do”): Qualitative Exploration of Clinicians’ and Service Providers’ Perspectives
Source: J Med Internet Res. 2024 Jan 29;26:e51245. doi: 10.2196/51245 (PMC10862246; doi:10.2196/51245)
Supplement: Multimedia Appendix 1 [file jmir_v26i1e51245_app1.docx]

## Appendix 1

Qualitative Interview Schedule

1. **Scam incidents with clients with brain injury**

- How many clients with ABI can you think of who have been scammed?
- What sorts of scams were they?
- Selecting the most significant scam incident, tell me about what happened
- What were the consequences of the scam for the person? What was the main psychological impact of the scam?
- How did you identify the psychological impacts of the scam? (measures, interview, observation, etc.)?
- Tell me about how you found out your client was scammed?
- How or by whom was the person with ABI told they had been scammed and how did they react?
- What was the client’s understanding of the scam?
- How did the family/others respond to finding out?
- Was it reported to any agency (e.g. police, scam watch, funder)?
- Did the person have a financial administrator or documented reduced capacity?
- What was done to help? What supports did you try? How did this go? What helped? What didn't help?
- Did the scam end – how?
- Were they scammed again? Tell me more…
- How do you think the ABI impacted their vulnerability to or recovery from the scam?
- What other factors increased their vulnerability (e.g. social isolation, easy going, flatness in affect trusting, enjoys social contact)?
- What would you do differently next time?
- Anything else you could tell me about the scam or the aftermath?

1. **Prevention**

- Can you tell me about any strategies or approaches you use (before or after the scam) to prevent your clients getting scammed?

1. **Resources**

- Did you have or did you look for any resources/supervision - what did you find/learn?
- What would help you increase your skills and capacity to respond? (e.g. resources, knowledge, supervision, time)

1. **Interventions**

- What strategies did you use to address psychological impacts (e.g. shame, embarrassment, distress)? (ABI-specific modifications or approaches)?
  - If no psychological interventions were delivered, why not?
  - If non-psych clinician: tell me about the interventions that were delivered.
- Were other professionals involved in the interventions? (types roles)
- How did they respond to the intervention?
- What factors impacted how the person with ABI engaged in the intervention? (facilitators, challenges)
- How do you think the ABI may affect their response to intervention?
- Tell me your thoughts on what the focus of future cybersafety recovery interventions should be (e.g. psychological, financial, digital literacy, education, scam risk prevention, etc.).
- What are your thoughts on the use of CyberAbility resources (preventive and e-safety strategies)?
- Tell me your thoughts and recommendations on the optimal delivery of future cybercrime recovery interventions.
  - Prompts: intervention structure (e.g. modular, flexible; group/individual/both, type of sessions for group/individual, intervention & session duration); involvement of others (e.g. other professionals, family members, close others – separate session?; how involved?)
- What topics would be helpful to be included in a structured intervention?
- What are your thoughts on a topic or module based on ‘Story-telling’? i.e., sharing scam experiences. How do you think this module could look like?
- What might be useful in specific modules surrounding psychological impacts (e.g., shame, grief)?
- How do you think these modules may look like?
- What are some ways we can engage people who may minimise or not acknowledge their risk to engage with the intervention/prevention?
- Anything else you could tell me about the approaches we could take to address the psychological impacts?

1. **Confidence**

- How did you feel as a clinician in addressing this? In terms of identifying and assisting (rate capacity to identity and respond out of 1-10, respectively)
- What ways do you identify someone might be scammed or at risk?
- How do you raise your suspicions with someone? What ways reduce their potential sense of shame?
